# Association of type 2 diabetes with periodontitis and tooth loss in patients undergoing hemodialysis

**DOI:** 10.1371/journal.pone.0267494

**Published:** 2022-05-06

**Authors:** Risako Mikami, Koji Mizutani, Yusuke Matsuyama, Tomohito Gohda, Hiromichi Gotoh, Norio Aoyama, Takanori Matsuura, Daisuke Kido, Kohei Takeda, Natsumi Saito, Takeo Fujiwara, Yuichi Izumi, Takanori Iwata

**Affiliations:** 1 Department of Periodontology, Graduate School of Medical and Dental Sciences, Tokyo Medical and Dental University, Bunkyo, Tokyo, Japan; 2 Department of Global Health Promotion, Tokyo Medical and Dental University, Bunkyo, Tokyo, Japan; 3 Department of Nephrology, Juntendo University Faculty of Medicine, Bunkyo, Tokyo, Japan; 4 Department of Internal Medicine, Saiyu Soka Hospital, Matsubara, Soka, Saitama, Japan; 5 Division of Periodontology, Department of Oral Interdisciplinary Medicine, Graduate School of Dentistry, Kanagawa Dental University, Inaokacho, Yokosuka, Kanagawa, Japan; 6 Weintraub Center for Reconstructive Biotechnology, Division of Advanced Prosthodontics, UCLA School of Dentistry, Los Angeles, California, United States of America; 7 Department of Behavioral Dentistry, Graduate School of Medical and Dental Sciences, Tokyo Medical and Dental University, Bunkyo, Tokyo, Japan; 8 Tokyo Medical and Dental University, Bunkyo, Tokyo, Japan; 9 Oral Care Perio Center, Southern Tohoku Research Institute for Neuroscience Southern Tohoku General Hospital, Koriyama, Fukushima, Japan; All India Institute of Medical Sciences, INDIA

## Abstract

**Background:**

Limited evidence are available regarding the influence of diabetes on periodontitis in hemodialysis patients, although the association between diabetes and periodontal disease is well-known.

**Objective:**

This study aimed to investigate the influence of type 2 diabetes mellitus (T2D) and its control level on periodontal disease and the number of missing teeth in patients undergoing hemodialysis.

**Subjects and methods:**

A single-center cross-sectional study was conducted on 246 Japanese patients with end-stage renal disease undergoing hemodialysis. Comprehensive medical and dental examinations were performed. The association between severity of periodontitis and T2D was examined by multiple ordered logistic regression analysis. A multiple linear regression model was fitted to assess the association of periodontal probing depth (PPD) ≥4 mm and the number of missing teeth with T2D (n = 125). A subgroup analysis involving only the patients with T2D was performed to investigate the factors associated with missing teeth among them.

**Results:**

After adjusting for confounders, the classification of periodontitis severity was significantly advanced in patients with T2D (odds ratio: 1.64, 95% confidence interval [CI]: 1.02–2.65, p = 0.04). The proportion of PPD≥4 mm sites and the number of missing teeth was significantly associated with T2D (coefficient: 4.1 and 5.7, 95% CI: 0.2–8.0 and 3.4–8.0, p = 0.04 and <0.001, respectively). Subgroup analysis of T2D patients revealed that glycoalbumin levels (coefficient: 0.4, 95% CI: 0.03–0.80, p = 0.03), but not hemoglobin A1c levels (coefficient: 0.8, 95% CI: -1.0–2.7, p = 0.37), were significantly associated with the number of missing teeth.

**Conclusion:**

T2D was significantly associated with periodontitis and the number of missing teeth in hemodialysis patients. Moreover, it is first documented that poor glycemic control, as determined by glycoalbumin levels, was significantly associated with the number of missing teeth in hemodialysis patients with T2D.

## Introduction

The number of patients with end-stage renal disease (ESRD) has been increasing and reached 3.7 million in the world. Among them, 1430 per million individuals are receiving dialysis therapy [1]. Diabetes is a major cause of dialysis initiation; indeed, diabetic kidney disease accounts for approximately 45% of dialysis initiation in Japan [2]. Additionally, diabetes has been reported as a significant risk factor for mortality due to the increased risk of cardiovascular and infectious disease in hemodialysis patients [3, 4].

Previous studies have elucidated bidirectional associations between diabetes and periodontal disease [5]. Periodontal disease is an inflammatory disease caused by infection with periodontal pathogenic bacteria such as *Porphyromonas gingivalis* (*P*. *gingivalis*) and is known to enhance systemic inflammation [6, 7], which is accelerated in patients with diabetes [8, 9]. Diabetes adversely affects the onset and progression of periodontal disease [10–12] by modulating cellular responses [13–15], leading to increased inflammation [16, 17]. In addition, periodontitis is known as a major cause of tooth loss. Destruction of alveolar bone, connective tissue, and periodontal ligaments occurs with periodontal pocket formation, leading to tooth loss [18].

We hypothesized that, in hemodialysis patients whose immune system has been impaired [19], type 2 diabetes mellitus (T2D) may be a more serious risk factor for tooth loss subsequent to the progression of periodontitis. Previous cross-sectional studies [20, 21] have demonstrated that the number of missing teeth was greater in hemodialysis patients with diabetes than in those without. However, these studies used simple screening tests to evaluate periodontitis and failed to find a clear association between periodontitis and diabetes in hemodialysis patients. Moreover, these studies did not adjust for important confounders such as age and smoking [22]. To our knowledge, no reports show the differences in comprehensively examined periodontal status in hemodialysis patients according to their diabetic status. Therefore, the purpose of this study was to investigate the association between T2D and periodontal disease following comprehensive periodontal examinations in patients with ESRD undergoing hemodialysis and to elucidate the factors associated with the severity of periodontitis and the number of missing teeth in patients with T2D using multivariate analysis.

## Materials and methods

### Study participants

A cross-sectional study was conducted using the data of patients with ESRD undergoing hemodialysis in one outpatient clinic in the Japanese Capital Region in April 2015 [23]. All participants provided written informed consent. This study was approved by the Research Ethics Committee of Tokyo Medical and Dental University (D2014-126) and was conducted in accordance with the Helsinki Declaration (as revised in Fortaleza, Brazil, October 2013). This study was registered in the University Hospital Medical Information Network (UMIN) Clinical Trials Registry (UMIN000039845). Individuals were included in the study if they were ≥20 years old and provided written informed consent. Those with acute inflammation were excluded. Patient information such as the history of diabetes, type of diabetes was obtained from medical records [24]. Diabetes mellitus was diagnosed by the physician according to the criteria of the Japan Diabetes Society [25]. Patients who had been diagnosed with T2D before the examination day were included in the T2D group. Measurement of body mass index (BMI), blood pressure, and blood biochemistry tests, including high sensitivity C-reactive protein (hsCRP), were performed.

### Dental examination and study outcomes

Periodontal probing depth (PPD) and bleeding on probing (BOP) were assessed using a manual probe (PCP-UNC 15, Hu-Friedy, Chicago, IL) at six sites on all remaining teeth by three experienced periodontists (KM, NA, and TM) trained for inter-rater reliability [26]. The examination was calibrated to reduce intra-examiner error and to reach reliability and consistency before the start of the study. Intraclass correlation coefficients (ICC) of inter-examiner agreement showed agreements of 0.87 (95% confidence intervals [CI]: 0.66–0.94). Intra-examiner agreement was calculated with ICC and showed an agreement of 0.90, 0.92 and 0.94 (95% CI: 0.86–0.97, 0.78–0.96, and 0.81–0.96), respectively for KM, NA, and TM. The severity of periodontal disease was classified into five categories (healthy, mild periodontitis, moderate periodontitis, severe periodontitis, edentulous) according to the modified Centers for Disease Control and Prevention and American Academy of Periodontology (CDC/AAP) case definition [27] (S1 Table). Clinical attachment level was measured for the diagnosis of the CDC/AAP case definition. To evaluate dental health, the number of decayed, missing, and filled teeth were recorded following the oral health assessment criteria by WHO as the DMFT index [28]. Oral hygiene status was evaluated according to debris index-simplified (DI-S) [29].

### Statistical analysis

Continuous variables are summarized as mean values and standard deviations (SD), and categorical variables are summarized as numbers and percentages. The characteristics of the subjects were compared between ESRD patients with and without T2D using t-tests and Fisher’s exact test. Multiple ordered logistic regression analysis was performed to investigate the association between diabetes and the severity of periodontitis. Multiple linear regression analysis was performed to investigate the association between diabetes and the proportion of sites with PPD ≥4 mm and the number of missing teeth. Participants with no remaining teeth (n = 38) were excluded when evaluating the association of T2D with the proportion of sites with PPD ≥4 mm. The analysis was adjusted for age, sex, BMI, smoking status, albumin, and hsCRP. We also performed a subgroup analysis involving only the patients with T2D, to investigate the factors associated with the number of missing teeth in this population. A p-value <0.05 was considered reflective of statistical significance. Statistical analyses were performed using STATA software (STATA software, version 15.0, Stata, College Station, TX).

## Results

**Fig 1:**
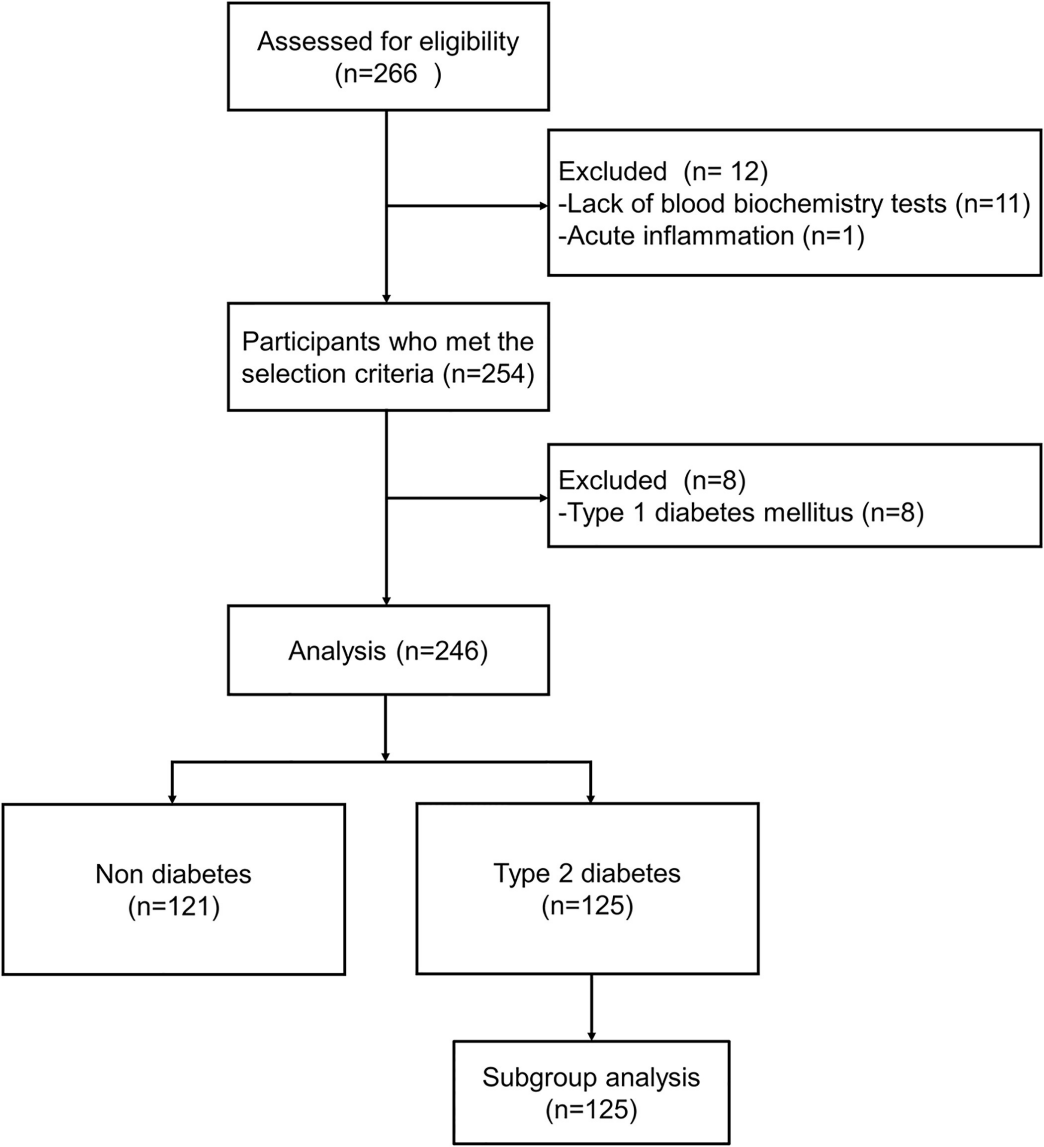
Flow diagram of the subject enrollment process.

A total of 266 patients participated in this study. Among those, 11 patients who lacked blood biochemistry tests, one patient with acute inflammation, and eight patients with type 1 diabetes mellitus were excluded (Fig 1). In total, 246 patients were included in this study (mean age [standard deviation] = 67.9 [11.8] years, male = 65.9%). The mean hemodialysis vintage was 80.7 months. The reasons for hemodialysis initiation were diabetic kidney disease (45.9%), nephrosclerosis (22.3%), chronic glomerulonephritis (20.7%), polycystic kidney disease (2.8%), and others (8.1%). Table 1 shows the demographic characteristics of study participants. Of the 246 participants, 51% had diabetes, with a mean disease duration of 18.7 years. The levels of HbA1c, fasting casual glucose, and glycoalbumin in patients with T2D were 6.5%, 141.7 mg/dL, and 20.4%, respectively. Older age, male sex, a history of smoking, and higher hsCRP levels were significantly more common in the T2D group than in the non-T2D group.

**Table 1:** Characteristics of the study participants.

		Non-diabetes	Type 2 Diabetes	
		(n = 121)	(n = 125)	
Characteristic		n, % or	n, % or	p-value
		mean ± SD	mean ± SD	
Male		70 57.8%	92 73.6%	0.01
Age (year)		66.0 ± 13.4	69.7 ± 9.6	0.02
Smoker	Never	59 48.8%	35 28.0%	0.002
	Former	43 35.5%	70 56.0%	
	Current	19 15.7%	20 16.0%	
BMI (kg/m^2^)	<18.5	28 23.1%	16 12.8%	0.11
	18.5–24.9	77 63.6%	91 72.8%	
	25≤	16 13.2%	18 14.4%	
Hemodialysis vintage (Mo)		93.4 ± 83.3	68.4 ± 58.3	0.01
Original disease	Diabetic kidney disease	0 0%	113 90.4%	<0.001
	Nephrosclerosis	49 40.5%	6 4.8%	
	Chronic glomerulonephritis	47 38.8%	4 3.2%	
	Polycystic kidney disease	7 5.8%	0 0%	
	Others	18 14.9%	2 1.6%	
Duration of diabetes (yrs)		-	18.7 ± 9.5	-
Casual blood glucose (mg/dL)		-	141.7 ± 43.8	-
HbA1c (%)		-	6.5 ± 1.0	-
Glycoalbumin (%)		-	20.4 ± 4.8	-
Prior CVD		15 12.4%	46 36.5%	<0.001
Systolic BP (mmHg)		145.9 ± 22.9	150.6 ± 26.1	0.14
Diastolic BP (mmHg)		80.5 ± 14.1	75.0 ± 15.7	0.004
White blood cell (10^3^/μL)		5.5 ± 1.4	6.8 ± 2.4	<0.001
Hemoglobin (g/dL)		10.8 ± 1.3	11.2 ± 2.5	0.11
Hematocrit (%)		33.2 ± 2.8	33.3 ± 4.1	0.87
Albumin (g/dL)		3.5 ± 0.4	3.4 ± 0.3	0.83
Blood urea nitrogen (mg/dL)		67.4 ± 16.8	61.6 ± 17.1	0.03
Creatinine (U/L)		10.7 ± 2.3	10.7 ± 7.0	0.93
corrected Calcium (mg/dL)*		9.2 ± 0.7	9.1 ± 0.7	0.45
Potassium (mg/dL)		5.3 ± 1.5	5.6 ± 1.6	0.15
eGFR (ml/min/1.73m^2^)		4.1 ± 1.3	4.6 ± 1.9	0.02
hsCRP (mg/dL)		0.43 ± 0.93	0.49 ± 0.75	0.01
**Dental health status**				
Number of decayed teeth		0.7 ± 1.7	1.1 ± 2.0	0.08
Number of missing teeth		8.3 ± 8.9	15.0 ± 9.7	<0.001
Number of filled teeth		7.7 ± 5.5	6.0 ± 5.8	0.01
DI-S		0.8 ± 0.1	1.2 ± 0.1	0.002
Severity of periodontitis	Healthy	7 5.8%	7 5.6%	0.01
	Mild periodontitis	38 31.4%	28 22.4%	
	Moderate periodontitis	48 39.6%	35 28.0%	
	Severe periodontitis	18 14.9%	27 21.6%	
	Edentulous	10 8.2%	28 22.4%	
PPD≥4mm (%)		6.6 ± 11.6	11.2 ± 15.5	0.02
PPD≤3mm (%)		93.4 ± 11.6	89.1 ± 15.3	0.02
BOP (%)		13.2 ± 16.2	15.3 ± 15.7	0.35

SD, Standard deviation; BMI, Body mass index; CVD, Cerebrovascular disease; HbA1c, Hemoglobin A1c; BP, Blood pressure; eGFR, Estimated glomerular filtration rate; hsCRP, High sensitivity c-reactive protein; DI-S, Debris index-simplified; PPD, Periodontal probing depth; BOP, Bleeding on probing.

* (corrected calcium (mg/dL)) = (total calcium (mg/dL)) + [4 − (serum albumin (g/dL))]

Regarding periodontal diagnosis, 14 (5.7%), 66 (26.8%), 83 (33.7%), 45 (18.3%) and 38 (15.4%) participants were classified into healthy, mild, moderate, severe periodontitis, and edentulous, respectively. In univariate analysis, patients with T2D had more advanced periodontal disease and a higher proportion of sites with PPD ≥4 mm (11.2 ± 15.5%) than those without diabetes (6.6 ± 11.6%). There were no significant differences in the proportion of BOP-positive sites between patients with and without diabetes. Furthermore, patients with T2D had lost 15.0 teeth on average, which was significantly more than those without diabetes, who had lost 8.3 teeth on average.

Table 2 shows the association of T2D with the severity of periodontitis. After adjusting for age, sex, BMI, smoking habits, serum albumin, and hsCRP, patients with T2D had a significantly higher odds ratio (OR) of disease progression in periodontitis than did those without diabetes (OR = 1.64, 95% CI: 1.02–2.65, p = 0.04) (Table 2). This analysis also confirmed that smoking, regardless of whether the patient is a former or current smoker, is associated with severe periodontal disease (former smoker: OR = 1.91, 95% CI: 1.04–3.49, p = 0.04; current smoker: OR = 2.62, 95% CI: 1.22–5.62, p = 0.01).

**Table 2:** Univariate and multivariate ordered logistic regression analysis of the factors influencing the severity of periodontitis in the study patients (n = 246).

		Crude model			Multivariate model†		
Covariates		OR	95% CI	p-value	OR	95% CI	p-value
Type 2 diabetes		1.98	1.25, 3.12	0.003	1.64	1.02, 2.65	0.04
Age (year)		1.03	1.01, 1.05	0.002	1.03	1.01, 1.05	0.01
Female		0.72	0.45, 1.17	0.19	1.01	0.61, 1.96	0.76
BMI (kg/m^2^)	<18.5	1.12	0.61, 2.04	0.71	1.28	0.68, 2.40	0.44
	18.5–24.9	ref.	-	-	ref.	-	-
	25≤	1.05	0.52, 2.07	0.90	0.98	0.49, 1.95	0.95
Smoking	Never	ref.	-	-	ref.	-	-
	Former	1.92	1.16, 3.18	0.01	1.91	1.04, 3.49	0.04
	Current	2.70	1.34, 5.43	0.01	2.62	1.22, 5.62	0.01
Albumin (g/dL)		0.80	0.40, 1.60	0.52	0.66	0.30, 1.42	0.29
hsCRP (mg/dL)		0.96	0.72, 1.27	0.76	0.87	0.64, 1.18	0.37

OR, Odds ratio; CI, Confidence interval; ref, Reference; BMI, Body mass index; hsCRP, High sensitivity c-reactive protein.

^†^Adjusted for all covariates.

To assess the impact of diabetes on periodontitis in hemodialysis patients, we analyzed the association between the proportion of sites with PPD ≥4 mm and T2D (Table 3). After adjusting for all covariates, patients with T2D had a significantly higher proportion of sites with PPD ≥4 mm than those without diabetes did (coefficient [coef.] = 4.1; 95% CI: 0.2–8.0; p = 0.04).

**Table 3:** Univariate and multivariate regression analysis of the factors influencing the proportion of sites (%) with PPD more than 4 mm in the study patients (n = 208).

		Crude model			Multivariate model†		
Variables		Coef.	95% CI	p-value	Coef.	95% CI	p-value
Type 2 diabetes		4.5	0.8, 8.2	0.02	4.1	0.2, 8.0	0.04
Age (year)		0.0	-0.1, 0.2	0.71	0.0	-0.2, 0.2	0.87
Female		-0.8	-4.7, 3.1	0.69	1.7	-3.0, 6.4	0.48
BMI (kg/m^2^)	<18.5	0.6	-4.4, 5.6	0.82	1.1	4.1, 6.2	0.68
	18.5–24.9	ref.	-	-	ref.	-	-
	25≤	1.1	-4.4, 6.6	0.70	0.7	-5.0, 6.3	0.82
Smoking	Never	ref.	-	-	ref.	-	-
	Former	3.6	-0.4, 7.6	0.08	3.5	-1.3, 8.3	0.15
	Current	4.6	-1.1, 10.3	0.11	5.4	-0.9, 11.7	0.10
Albumin (g/dL)		-3.1	-8.3, 2.1	0.24	-2.3	-8.6, 4.0	0.48
hsCRP (mg/dL)		1.3	-0.8, 3.4	0.23	0.7	-1.8, 3.1	0.59

Coef, Coefficient; CI, Confidence interval; ref, Reference; BMI, Body mass index; hsCRP, High sensitivity c-reactive protein.

^†^Adjusted for all covariates.

Table 4 shows the association of T2D with the number of missing teeth. Patients with T2D had lost significantly more teeth than had those without diabetes after adjusting for all covariates (coef. = 5.7, 95% CI: 3.4–8.0, p < 0.001) (Table 4).

**Table 4:** Univariate and multivariate regression analysis of the factors influencing the number of missing teeth in the study patients (n = 246).

		Crude model			Multivariate model†		
Covariates		Coef.	95% CI	p-value	Coef.	95% CI	p-value
Type 2 diabetes		6.6	4.3, 9.0	<0.001	5.7	3.4, 8.0	<0.001
Age (year)		0.3	0.2, 0.4	<0.001	0.3	0.2, 0.4	<0.001
Female		-0.9	-3.5, 1.7	0.51	1.1	-1.6, 3.9	0.42
BMI (kg/m^2^)	<18.5	1.2	-2.1, 4.5	0.47	2.1	-0.9, 5.0	0.17
	18.5–24.9	ref.	-	-	ref.	-	-
	25≤	-1.3	-5.0, 2.3	0.48	0.6	-2.7, 3.9	0.70
Smoking	Never	ref.	-	-	ref.	-	-
	Former	1.7	-1.0, 4.4	0.23	1.0	-1.8, 3.8	0.49
	Current	1.3	-2.4, 5.0	0.49	3.4	0.2, 7.0	0.06
Albumin (g/dL)		-5.4	-8.8, -2.0	0.002	-2.6	-6.2, 1.0	0.16
hsCRP (mg/dL)		0.2	01.3, 1.7	0.78	-0.6	-2.1, 0.8	0.39

Coef, Coefficient; CI, Confidence interval; ref, Reference; BMI, Body mass index; hsCRP, High sensitivity c-reactive protein.

^†^Adjusted for all covariates.

To investigate the factors influencing tooth loss in patients with T2D, the associations between variables related to diabetes and the number of missing teeth were assessed. The analysis showed that glycoalbumin (coef. = 0.4, 95% CI: 0.03–0.8, p = 0.03) had a significant association with the number of missing teeth after adjustment for the duration of diabetes, age, sex, BMI, smoking habits, serum albumin, and hsCRP (Table 5 model 1). Furthermore, current smoking was found to be significantly associated with increased missing teeth in patients with T2D (coef. = 6.4, 95% CI: 0.4–12.4, p = 0.04) in this model. On the contrary, the HbA1c level had no significant association with the number of missing teeth after adjusting for all covariates (Table 5 model 2).

**Table 5:** The factors associated with the number of missing teeth in patients with type 2 diabetes mellitus (n = 125).

		Crude model			Multivariate					
					Model 1†			Model 2†		
Covariates		Coef.	95% CI	p-value	Coef.	95% CI	p-value	Coef.	95% CI	p-value
Glycoalbumin (%)		0.3	-0.04, 0.7	0.09	0.4	0.03, 0.8	0.03			
HbA1c (%)		-0.2	2.0, 1.6	0.86				0.8	-1.0, 2.7	0.37
Duration of diabetes (year)		0.0	-0.1, 0.2	0.69	-0.1	-0.3, 0.1	0.43	0.0	-0.2, 0.1	0.67
Age (year)		0.3	0.2, 0.5	<0.001	0.4	0.2, 0.6	<0.001	0.4	0.2, 0.6	<0.001
Female		1.5	-2.4, 5.4	0.45	3.7	-0.9, 8.3	0.11	3.3	-1.4, 7.9	0.17
BMI (kg/m^2^)	<18.5	4.5	-0.7, 9.7	0.09	3.3	-2.0, 8.6	0.22	4.0	-1.4, 9.3	0.15
	18.5–24.9	ref.	-	-	ref.	-	-	ref.	-	-
	25≤	-2.0	-6.9, 3.0	0.43	1.5	-3.5, 6.5	0.55	0.8	-4.2, 5.9	0.74
Smoking	Never	ref.	-	-	ref.	-	-	ref.	-	-
	Former	-0.9	-4.9, 3.1	0.67	2.3	-2.4, 7.0	0.33	2.1	-2.7, 6.9	0.38
	Current	0.9	-4.5, 6.4	0.73	6.4	0.4, 12.4	0.04	5.1	-0.9, 11.1	0.09
Albumin (g/dL)		-4.36	-9.5, 0.8	0.096	-1.2	-6.6, 4.3	0.67	-1.5	-7.0, 4.1	0.60
hsCRP (mg/dL)		0.3	-2.0, 2.6	0.80	-0.2	-2.5, 2.2	0.89	-0.1	-2.6, 2.3	0.91

Coef, Coefficient; CI, Confidence interval; HbA1c, Hemoglobin A1c; ref, Reference; BMI, Body mass index; hsCRP, High sensitivity c-reactive protein.

^†^Adjusted for all covariates.

## Discussion

To the best of our knowledge, this is the first report to show a significant association of T2D with both periodontal disease and the number of missing teeth in ESRD patients undergoing hemodialysis after adjusting for relevant confounders. Hemodialysis patients with T2D suffered more severe periodontitis and lost a greater number of teeth than those without diabetes, although the proportion of BOP-positive sites did not differ. In participants with T2D, especially, we demonstrated that poor glycemic control was associated with tooth loss.

We found that the periodontal disease classification based on PPD was more statistically severe in hemodialysis patients with diabetes than in those without diabetes. The association between diabetes and periodontal status in this study was not consistent with the findings of previous studies of hemodialysis patients. Teratani et al. compared the periodontal parameters of 98 Japanese dialysis patients with and without diabetes [20]. They found no significant difference in the severity of periodontitis as indicated by BOP-positive sites or deep periodontal pockets. Naruishi et al. reported that, among 164 Japanese patients with chronic kidney disease, there was no difference in alveolar bone loss or the severity of periodontitis [21]. There are several possible reasons for such discrepancies between the results of the present and previous studies. The first possible reason is the difference in the evaluation method for periodontal disease. In the previous studies, periodontal disease was examined with a simple oral screening test, while herein, the periodontal examinations were more thorough and included measurements of the PPD and BOP at six sites in each tooth in all residual teeth. Therefore, we may have been able to evaluate periodontal disease more accurately and to find the influence of diabetes on periodontitis. Second, the number of participants in the present study was relatively large, and the age distribution of the participants was higher than that in previous studies. In the future, prospective and large-scale studies on hemodialysis patients will be required to determine the effect of diabetes on the progression of periodontal disease.

Previous studies have suggested that diabetes affects tooth loss in hemodialysis patients [20, 21]; however, these studies did not adjust for important confounders such as age and smoking [22]. In the present study, tooth loss was also associated with diabetes, but the relationship weakened after adjusting for relevant confounding factors. In the elderly population, tooth loss is mainly caused by periodontitis, but dental caries and tooth fracture can lead to tooth extraction. The patients with T2D in the present study had lost 15 teeth on average, which is a substantially greater number than that in the age-matched general population [30]. In addition, the factors associated with tooth loss in T2D participants were assessed in this study. We found that, although the number of missing teeth was not significantly associated with the duration of diabetes and the HbA1c level, it had a significant association with glycoalbumin. Because of the increases in the percentage of juvenile erythrocytes due to the effects of shortened erythroid lifespan, renal anemia, and the administration of erythropoiesis-stimulating agents, HbA1c is lower than the actual level of glycemic control in hemodialysis patients, and glycoalbumin reflects the glycemic control more precisely than does HbA1c [31, 32]. Therefore, the association of the number of missing teeth with glycoalbumin that we identified herein indicates that poor glycemic control may be related to increased tooth loss in hemodialysis patients with T2D.

It is still controversial whether diabetes affects BOP in the general population [33]. In this study, we found no difference in the ratio of BOP-positive sites between patients with T2D and those without diabetes. This result is in agreement with that of Teratani et al [20], and indicates that the ESRD state itself, but not the diabetic state, might be a crucial factor for chronic inflammation. Since BOP is an index reflecting the local gingival inflammation, it was suggested that diabetes may significantly modify the tissue destruction that occurs following the inflammation. Diabetes has been known to affect cells in periodontal tissue, such as fibroblasts [34, 35], osteoblasts, and osteoclasts, to enhance the destruction of periodontal tissue that accompanies inflammation [36, 37]. Diabetes might lead to the progression of periodontitis by modifying the host response to local inflammation [38].

Smoking was shown to be associated with the severity of periodontitis and tooth loss in this study. From numerous previous reports, smoking is known to be a strong risk factor for periodontitis [39] and tooth loss [22] in the general population. The results of the present study support those of previous reports and suggest that smoking is associated with severe periodontitis and tooth loss in hemodialysis patients.

This study has several limitations. Firstly, this was a cross-sectional study; the causality, therefore, remains unknown. Further prospective studies elucidating the relationship between diabetes and periodontal disease in hemodialysis patients are required. Second, the patients of the present study were all Japanese hemodialysis patients from one hospital. Further studies conducted in different regions or races are needed to confirm if the results of this study are applicable to other populations. Thirdly, although we performed multiple regression analyses, there may be residual confounding factors such as socioeconomic status that were not adjusted for in this study. Finally, various underlying diseases for ESRD could not be investigated separately due to the small number of patients. Since the underlying disease that led to hemodialysis varied, especially in the non-diabetes group, stratified analysis by primary disease might be meaningful if the study had a sufficient number of participants.

## Conclusion

In conclusion, T2D was associated with severe periodontal disease and the number of missing teeth in patients with ESRD undergoing hemodialysis. Moreover, poor glycemic control was associated with the number of missing teeth among those with T2D. This study showed that patients with T2D are more likely to suffer poor oral health among patients undergoing hemodialysis. Therefore, it is necessary to take measures to improve their oral health, such as having medical practitioners recommend a dental examination and dental care to the patients under hemodialysis maintenance.

## Supporting information

S1 Table The severity of periodontitis used in this study. (DOCX)

S1 Checklist (DOCX)

S2 Checklist (PDF)
