# Supplementary material for: Association of type 2 diabetes with periodontitis and tooth loss in patients undergoing hemodialysis
Source: PLoS One. 2022 May 6;17(5):e0267494. doi: 10.1371/journal.pone.0267494 (PMC9075673; doi:10.1371/journal.pone.0267494)
Supplement: S1 Table — (DOCX) [file pone.0267494.s001.docx]

**S1 Table**

| Supplementary table 1. The severity of periodontitis used in this study*. | | | |
| --- | --- | --- | --- |
|  | **Clinical Definition** | | |
| **Disease Category** | CAL |  | PPD |
| Healthy | ≤ 1 interproximal sites with CAL ≥ 3 mm | and | ≤ 1 interproximal sites with PPD ≥ 4 mm |
| Mild periodontitis | ≥ 2 interproximal sites with CAL ≥ 3 mm (not on same tooth) | or | ≥ 2 interproximal sites with PPD ≥ 4 mm (not on same tooth) |
| Moderate periodontitis | ≥ 2 interproximal sites with CAL ≥ 4 mm (not on same tooth) | or | ≥ 2 interproximal sites with PPD ≥ 5 mm (not on same tooth) |
| Severe periodontitis | ≥ 2 interproximal sites with CAL ≥ 6 mm (not on same tooth) | and | ≥ 1 interproximal sites with PPD ≥ 6 mm |
| Edentulous | No residual teeth in the oral cavity | | |
| CAL, Clinical attachment level; PPD, Periodontal probing depth. | | | |
| *modified with the Centers for Disease Control and Prevention and American Academy of Periodontology case definition (Page *et al.* 2007). | | | |
