# Supplementary material for: Association of type 2 diabetes with periodontitis and tooth loss in patients undergoing hemodialysis
Source: PLoS One. 2022 May 6;17(5):e0267494. doi: 10.1371/journal.pone.0267494 (PMC9075673; doi:10.1371/journal.pone.0267494)
Supplement: S1 Checklist — (DOCX) [file pone.0267494.s002.docx]

NEWCASTLE - OTTAWA QUALITY ASSESSMENT SCALE

**(adapted for cross sectional studies)**

This scale has been adapted from the Newcastle-Ottawa Quality Assessment Scale for cohort studies and the adapted Newcastle-Ottawa scale created for cross-sectional studies by Modesti et al.^1^.

**Selection:** (Maximum 5 stars)

****

1. Representativeness of the sample:
   1. Truly representative of the average in the target population. * (all subjects or random sampling)
   2. Somewhat representative of the average in the target population. * (non- random sampling)
   3. Selected group of users.
   4. No description of the sampling strategy.
2. Sample size:
   1. Justified and satisfactory. *
   2. Not justified.
3. Non-respondents:
   1. Comparability between respondents and non-respondents characteristics is established, and the response rate is satisfactory. *
   2. The response rate is unsatisfactory, or the comparability between respondents and non-respondents is unsatisfactory.
   3. No description of the response rate or the characteristics of the responders and the non-responders.
4. Ascertainment of the exposure (risk factor):
   1. Validated measurement tool. **
   2. Non-validated measurement tool, but the tool is available or described.*
   3. No description of the measurement tool.

**Comparability:** (Maximum 2 stars)

**

1. The subjects in different outcome groups are comparable, based on the study design or analysis. Confounding factors are controlled.
   1. The study controls for the most important factor (select one). *
   2. The study control for any additional factor. *

**Outcome:** (Maximum 3 stars)

***

1. Assessment of the outcome:
   1. Independent blind assessment. **
   2. Record linkage. **
   3. Self report. *
   4. No description.
2. Statistical test:
   1. The statistical test used to analyze the data is clearly described and appropriate, and the measurement of the association is presented, including confidence intervals and the probability level (p value). *
   2. The statistical test is not appropriate, not described or incomplete.

1. Modesti PA, Reboldi G, Cappuccio FP, et al. Panethnic Differences in Blood Pressure in Europe: A Systematic Review and Meta-Analysis. *PLoS One.* 2016;11(1):e0147601.
